# Implementing digital mental health interventions at scale: one-year evaluation of a national digital CBT service in Ireland

**DOI:** 10.1186/s13033-023-00592-9

**Published:** 2023-10-10

**Authors:** Siobhán Harty, Angel Enrique, Selin Akkol-Solakoglu, Adedeji Adegoke, Hannah Farrell, Graham Connon, Fiona Ward, Conor Kennedy, Derek Chambers, Derek Richards

**Affiliations:** 1 Amwell Science, Amwell, One Stephen Street Upper, Dublin 8, Dublin, Ireland; 2 Amwell Clinical Operations, Amwell, One Stephen Street Upper, Dublin 8, Dublin, Ireland; 3 Community Health Organization, Health Service Executive (HSE), Dublin, Ireland; 4 National Counselling Service, Health Service Executive (HSE), Dublin, Ireland; 5 Health Service Executive (HSE), National Mental Health Operations, Dublin, Ireland

**Keywords:** Digital, Cognitive behavioral therapy, Depression, Anxiety, National Health Service

## Abstract

**Background:**

In recent years, exponential growth in digital innovations and internet access has provided opportunities to deliver health services at a much greater scale than previously possible. Evidence-based technology-enabled interventions can provide cost-effective, accessible, and resource-efficient solutions for addressing mental health issues. This study evaluated the first year of a supported digital cognitive behavioral therapy (CBT) service provided by the national health service in Ireland, which has been accessible to individuals who receive a referral from one of five referring groups: General Practitioners, Primary Care Psychology, Counselling Primary Care, Community Mental Health, and Jigsaw (a nationwide youth mental health service).

**Methods:**

A retrospective, observational study examining data from the service between April 2021 to April 2022 was conducted. Descriptive statistics on referrals, account activations, user demographics, program usage, and user satisfaction were extracted, and pre-to-post clinical outcomes for depression measured by the Patient Health Questionnaire-9 and for anxiety measured by the Generalised Anxiety Disorder-7 were analysed using linear mixed effect models.

**Results:**

There were 5,298 referrals and 3,236 (61%) account activations within the year. Most users were female (72.9%) and aged between 18 and 44 years (75.4%). The CBT programs were associated with significant reductions in both depression (β = 3.34, 95% CI [3.03, 3.65], p < 0.001) and anxiety (β = 3.64, 95% CI [3.36, 3.93], p < 0.001), with large effect sizes (Cohen’s d > 0.8). Time spent using the programs was also found to be a predictor of the variability in these clinical outcomes (p < 0.001), and accounting for this resulted in significantly better model fits (p < 0.001). User satisfaction ratings were also very high, exceeding 94%.

**Conclusions:**

Efforts to improve the representation of male and older adult users are warranted. However, overall, the results demonstrate how digital CBT can be provided at scale and lead to symptom reductions with large effect sizes for patients seeking help for depression and anxiety. The findings substantiate the continued use and expansion of this service in Ireland and the more widespread implementation of similar services in other international public healthcare settings.

**Supplementary Information:**

The online version contains supplementary material available at 10.1186/s13033-023-00592-9.

## Introduction

Depression and anxiety disorders are the two leading causes of health-related burdens globally [1]. According to the World Health Organization [2], 25% of the population of Europe suffers from depression or anxiety disorders each year. In Ireland, recent estimates have suggested that 23–30% of adults have a major depressive disorder, and 20–22% have a generalized anxiety disorder [3]. These psychological disorders constitute significant psychosocial and economic challenges [4], and the ongoing COVID-19 pandemic has exacerbated their impact and prevalence [5].

In Ireland and internationally, General Practitioners (GPs) in primary care are usually the first formal point of contact for individuals seeking professional help for a mental health problem [6–9]. Resource issues in the primary care system often result in many individuals being left untreated [10, 11] or an overreliance on medication for treating depression and anxiety [9, 12, 13]. Improving patients’ access to evidence-based psychological therapies is crucial in public health services considering patients’ three-fold preference for psychological treatment over medication [14] and concerns about the adverse effects of long-term antidepressant use [15, 16].

Awareness of the ongoing mental healthcare access crisis has prompted governments, policymakers, and healthcare services to pilot digital health initiatives that provide novel and innovative ways of bridging the gap between patient needs and available mental health support [17, 18]. Digital cognitive behavioral therapy (CBT) has been widely recognized as a promising first-line treatment since it is easily accessible, cost-effective, resource-efficient [19, 20]. It also has a robust evidence base to support its effectiveness in treating depression and anxiety [21–26].

Digital CBT services are already part of routine care in some countries, operating as stand-alone interventions or in conjunction with traditional face-to-face care. For example, since 2008, digital CBT has been one of the available therapies in the UK’s Improving Access to Psychological Therapies (IAPT) services provided for adults with subthreshold depressive symptoms or mild to moderate depression and anxiety [27, 28]. Other examples of digital CBT programs successfully implemented within routine clinical practice have been described elsewhere [29–32]. These clinical services have provided effective treatments to many patients and successfully progressed from pilot trials to permanent services. Some key success factors of these services were using evidence-based CBT programs, monitoring patient progress, accepting self-referrals and referrals from health professionals, having efficient processes for conducting online and telephone assessments, and incorporating user experience and satisfaction feedback to improve service delivery.

In Ireland, the national health service, known as the Health Service Executive (HSE), provides a wide range of publicly funded community and hospital-based mental health services to address mental health problems, supported by multidisciplinary teams including psychiatrists and other mental health professionals. However, it has also been acknowledged that there are significant gaps in the provision and access to mental health services [17]. In 2021, following an initial pilot, the HSE partnered with SilverCloud Health (SCH), a provider of evidence-based digital behavioral healthcare solutions, to make mental health support more widely accessible for individuals experiencing depression and anxiety.

The present paper provides an overview of this national digital CBT service and a one-year milestone evaluation, focusing on service-level metrics such as referrals and account activations, user-level data such as user demographics, baseline symptomatology, clinical outcomes, program usage and user satisfaction.

## Methods

### Study Design and setting

We conducted a retrospective, observational study examining data from the digital CBT service provided through the national health service in Ireland between April 20^th,^ 2021 and April 19th, 2022. Referrals have come from GPs, Primary Care Psychology, Counselling in Primary Care, Community Mental Health, and Jigsaw (a charity providing a nationwide youth mental health service and receives funding from the HSE).

All procedures contributing to this study adhered to the ethical standards of the relevant national and institutional committees on human experimentation and with the Helsinki Declaration of 1975, as revised in 2008. The protocol related documents and informed consent have been reviewed and approved by the Trinity College Dublin School of Psychology Research Ethics Committee (Approval ID: SPREC112021-04).

### Referral and signup process

The HSE issued communications detailing the launch of the new nationwide digital CBT service to health professionals across the five referral groups. SilverCloud additionally provided information in the form of emails, flyers and posters to these health professionals, which explained who would be suitable for SilverCloud and provided details about how to have the referral conversation with their patients. This information was further consolidated through follow-up webinars and meetings organised by SilverCloud for referring health professionals.

When considering a client for SilverCloud, health professionals were required to conduct an initial screening to assess whether they may be suitable for the intervention. Clients are considered eligible if they are aged 18 or over and experiencing low mood, depression, anxiety, worry, feelings of isolation, stress, poor sleep, or COVID-related fatigue based on their self-report. Clients who report experiencing severe mental health symptoms such as manic or psychotic symptoms or express wishes and/or plans to end their life or harm others are not deemed eligible for the service. When a client is deemed suitable, the referring clinician submits a referral to SilverCloud, which triggers an email invite sent to the client through the SilverCloud platform. Weekly reminder emails are sent to clients for the first 30 days following the initial invitation to remind clients that have not yet activated their account that they still have the opportunity to do so. Once clients click on the invitation link in the email, they are directed to the SilverCloud landing page, where they can initiate the sign-up process. During the sign-up process, clients are asked to provide consent for their pseudoanonymized data to be used for clinical research and service evaluation purposes. At this stage, they are also asked to provide personal and demographic information and complete baseline clinical questionnaires (described below). Of note, the clinical questionnaires include an item which appraises suicidality (item-9 of the *Patient Health Questionnaire-9*), and the risk management protocol is triggered if a client expresses wishes and/or plans to end their life or harm others (for further details on the Risk Management protocol, see the Supplementary Materials). After completing these sign-up steps, users are directed to the homepage of the therapeutic program assigned to them.

### Support during the intervention

Once users complete the sign-up process, they are assigned a supporter who provides guidance and feedback based on the user’s progress and clinical symptomology. All supporters are graduate psychologists with at least a master’s degree or doctorate in progress who have been trained in the delivery of online support.

Supporter training involves tutorials on how to use the platform and education on supporting clients as they work their way through programmes. Supporters have access to these materials to recap at any time. They also receive regular supervision from the Clinical Supervisors and have access to continuing professional development opportunities. Supporters are also required to attend at least one group supervision monthly and 1:1 supervision every two months. They also have access to a Clinical Supervisor daily within the working week. These processes ensure adherence to professional standards for service delivery and facilitate professional development for supporters.

The feedback and guidance provided by supporters is termed a ‘review.’ Reviews are provided asynchronously in written format via a message users receive on the platform. The primary purpose of these reviews is to ensure that all users receive personalised support and guidance in applying the learning from the programmes to their daily lives. Users are encouraged to respond to these reviews and take advantage of the opportunity to ask questions and clarify their understanding of the programme content. Supporter reviews are provided weekly for the first six weeks. Additional reviews may be provided at a fortnightly cadence at the discretion of the supporter and clinical supervisor, amounting to a maximum of 10 reviews. Once all the reviews are provided, or if users do not engage during three consecutive review periods, supporters discharge users from the supported service, meaning the supporter ceases to offer reviews. After users are discharged, they still have full access to the platform in an unsupported capacity.

### Intervention and client experience

The digital CBT programs offered through this service to date have been the following: *Space from Depression*, *Space from Anxiety*, *Space from Depression and Anxiety*, and *Space from Generalised Anxiety Disorder*. The structure and content of each of these programs align with the evidence-based principles of face-to-face CBT. The programs aim to develop and increase an understanding of the relationship between one’s thoughts, feelings, and behaviours, alongside information about coping strategies and therapeutic techniques such as graded exposure, behavioral activation, cognitive restructuring, problem-solving and activity scheduling.

The program content is delivered online through the SilverCloud digital platform through various interactive media and tools, including quizzes, videos, activities, and personal stories. Further details on these programs’ therapeutic components, content, and efficacy can be found elsewhere [20, 24, 33].

During the course of the intervention, users complete brief clinical measures (Patient Health Questionnaire-2 and Generalised Anxiety Disorder-2) on a biweekly basis, and the Patient Health Questionnaire-9 (PHQ-9) Generalised Anxiety Disorder-7 (GAD-7) and Work and Social Adjustment Scale (WSAS) every 4 weeks, which are shared with the supporter. Risk is monitored continuously through item-9 on the PHQ-9 (for further details on the Risk Management protocol, see the Supplementary Materials).

### Primary outcome measures

*Patient Health Questionnaire-9 (PHQ-9)* is a nine-item self-report measure developed as a screening instrument for depression [34, 35]. Each item is rated on a scale of 0 to 3, with response options “not at all”, “several days”, “more than half the days”, and “nearly every day”. The total scale score ranges from 0 to 27, where cut-off points of 5, 10, 15, and 20 represent mild, moderate, moderately severe, and severe levels of depression, respectively. The PHQ-9 has good internal reliability, sensitivity, and specificity for major depression, with Cronbach’s alpha of 0.89, 0.88, and 0.88, respectively [34, 36].

*Generalised Anxiety Disorder (GAD-7)* is a 7-item self-report measure developed to identify individuals with a generalised anxiety disorder [37]. Each item is rated on a scale of 0 to 3, with response options “not at all”, “several days”, “more than half the days”, and “nearly every day”. The total scale score ranges from 0 to 21, where cut-off points of 5, 10, 15 represent mild, moderate, and severe anxiety levels, respectively. The GAD-7 has also demonstrated excellent internal reliability, good sensitivity, and specificity, with Cronbach’s alpha of 0.92, 0.89, and 0.82, respectively [37].

### Secondary measures

*Work and Social Adjustment Scale (WSAS)* is a 5-item self-report measure assessing an individual’s impairment and experiential impact across five life domains: work, social life, home life, private life, and close relationships [38]. The total scale scores range from 0 to 40, with higher scores indicating more significant functional impairment. The WSAS demonstrated high internal reliability, with a Cronbach’s alpha of 0.82, and a sensitivity to treatment effects comparable to widely used measures like the PHQ-9 and GAD-7 [39].

*Usage metrics* corresponding to the total time spent on the platform and the total number of logins were automatically recorded via the platform and were extracted for analysis.

*User Satisfaction* was also evaluated. At the end of each module, users can evaluate the module by rating the following four statements on a scale from “strongly disagree” to “strongly agree”: (1) The module was interesting; (2) The module was relevant; (3) The module was helpful; and (4) The program is helping me make progress. Users can rate as many of the statements as they want or skip the questionnaire entirely. The percentage of “agree” or “strongly agree” ratings are aggregated for each statement to provide an index of user satisfaction.

### Data Analysis

Descriptive statistics were used to evaluate all service level and user level variables. Linear mixed-effect models (LMM), with time as a fixed effect and service user as a random effect, were additionally used to determine whether there were significant changes in scores on the PHQ-9, GAD-7 and WSAS. These LMM were conducted on an intention-to-treat (ITT) basis, such that all discharged users who provided baseline data were included in the analyses, and maximum likelihood estimates were generated for users that did not complete follow-up clinical measures.

Given that program usage variables have the potential to shed light on the extent to which users engaged with the intervention [25], we additionally sought to determine whether the inclusion of program usage variables would improve the fit of these LMM. Unsurprisingly, there was high collinearity between ‘number of logins’ and ‘time spent on the platform’ (*r* = 0.8, *p* < .001). As such, we solely focused on comparing the models with and without the inclusion of ‘time spent on the platform’ as an independent predictor.

Effect sizes post-intervention were calculated using Cohen’s *d* formula, whereby the difference in the pre-to-post means for each clinical measure were divided by the pooled standard deviations of each of these measurement time points [40, 41]. The magnitude of these effect sizes was interpreted according to the benchmarks for small (*d* = 0.2), medium (*d* = 0.5), and large (*d* = 0.8) effects suggested [41].

For the primary outcome measures (PHQ-9 and GAD-7) rates of reliable improvement and recovery were also reported for discharged users that had completed at least one set of follow-up clinical measures. Consistent with previous research, reliable change indices (RCI) of 6-points and 4-points were used as criteria to measure reliable improvement and reliable deterioration on the PHQ-9 and GAD-7 respectively [38, 42, 43]. “Reliable improvement” is achieved when there is a decrease in score that is equal to or greater than the RCI of the measure, whereas the criterion for “reliable deterioration” is an increase in score that is equal to or greater than the RCI of the measure. A service user is considered to have achieved “recovery” if they transitioned from being at caseness prior to treatment to non-caseness post-treatment, where caseness is defined as scores ≥ 10 on the PHQ-9 and ≥ 8 on the GAD-7.

## Results

### Referrals and Account Activations

**Fig. 1:**
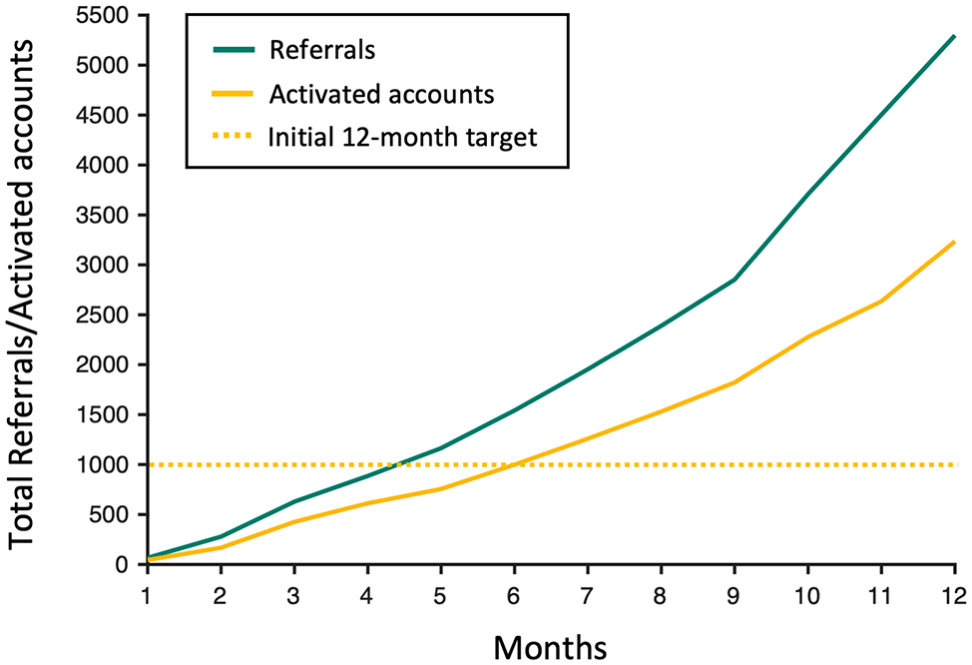
Total number of referrals and account activations within the first 12 months. The number of account activations exceeded the initial 12-month target (yellow dashed line) almost 6 months ahead of schedule

As shown in Fig. 1, the volume of referrals (i.e. invites to the platform) increased steadily over the course of the year, from a total of 629 referrals in the first 3 months (April 20th – July 19th 2021) to a total of 2446 referrals in the final 3 months (January 20th – April 19th 2022). The volume of account activations followed a similar pattern of escalation over the course of the year. However, the total number of account activations did not reach the total number of referrals at any time, reflecting how not all patients referred to the service followed through on activating accounts. The overall account activation rate was 61%.

The volume of referrals from each of the five referral sources, and the associated activation rates are shown in Table 1. General practitioners (GPs), the largest group of healthcare professionals in primary care in Ireland [8], have been the source of the majority of referrals.

The activation rates across referral sources range from 46 to 63.4%, with Primary Care Psychology accounting for the highest account activation rate. However, a chi-square test indicated that the differences in active activation rates across referral sources did not quite reach the threshold for statistical significance (*X*^*2*^ = 9.33, *p* = 0.053).

**Table 1:** Volume of referrals and activation rates across each of the five referral sources

Referring Group	Total Referrers	Median Referrals per Referrer (Range)	Total Referrals	Total Activated Accounts	Overall Activation Rate (%)
**General Practitioners**	993	2 (1–96)	4603	2818	61.2
**Primary Care Psychology**	43	2 (1–80)	268	170	63.4
**Counselling Primary Care**	34	2 (1–51	216	135	62.5
**Jigsaw**	37	2 (1–14)	124	73	58.9
**Community Mental Health**	22	1.5 (1–32)	87	40	46.0
**Total**	**1129**	**2 (1–96)**	**5298**	**3236**	**60.9**

### Baseline characteristics and Symptomatology

**Fig. 2:**
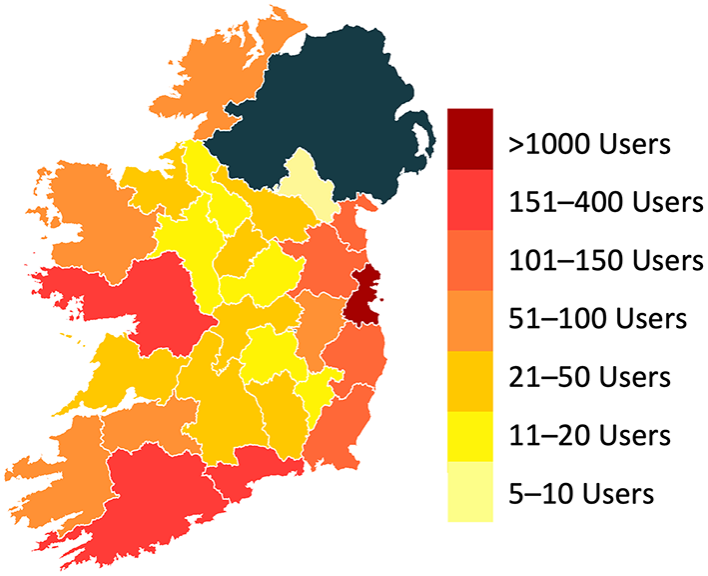
Distribution of users across the 26 counties of the Republic of Ireland. Warmer colors indicate a higher concentration of users

**Fig. 3:**
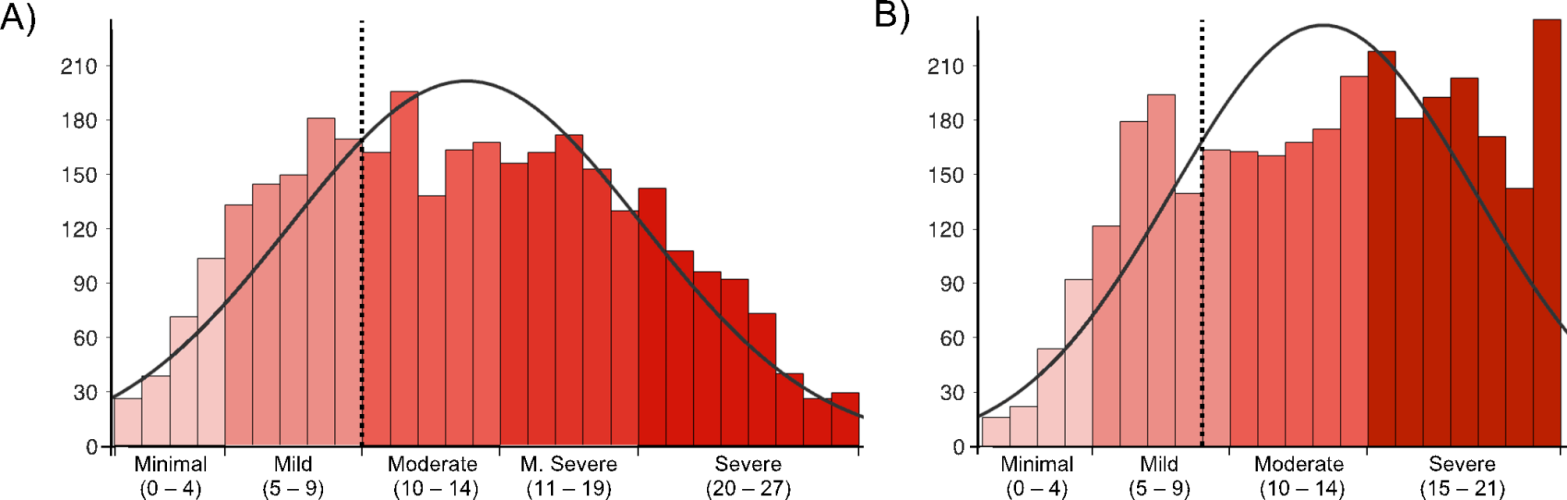
Baseline levels of depression and anxiety as measured by the PHQ-9 **(A)**, GAD-7 **(B)**, respectively. The dashed lines indicate the threshold for ‘caseness’ for each measure. M. = Moderately

Demographic information and other baseline characteristics of service users are presented in Table 2. Service users were primarily female, white Irish, well-educated, and aged between 18 and 44. There was a representation of service users from across all 26 counties of the Republic of Ireland, ranging from a maximum of 1064 from Dublin to a minimum of 7 from Monaghan (see Fig. 2). The most used program to date has been the ‘*Space from Anxiety*’ program, consistent with the prevalence of moderate-to-severe anxiety levels exhibited by the present sample (see Fig. 3). Expectations at baseline were generally positive, with the vast majority (90.1%) of users indicating that they believed SilverCloud digital CBT was at least “Somewhat likely” to work for them. The number of users that reported accessing concurrent psychological treatment was low (13.7%).

**Table 2:** Baseline service user characteristics

	Category	n (%*)
**Age**	18–24	754 (23.4%)
	25–34	869 (27.9%)
	35–44	775 (24.1%)
	45–54	503 (15.6%)
	55–64	200 (6.2%)
	65+	73 (2.3%)
**Gender**	Female	2345 (72.9%)
	Male	827 (25.7%)
	Other/Prefer not to say	25 (0.8%)
**Education**	College/University Degree	1722 (53.5%)
	Postgraduate Masters/Doctorate	411 (12.8%)
	Secondary Education	940 (29.2%)
	Primary Education	72 (2.2%)
**Ethnicity**	White Irish	2754 (85.6%)
	Other White European	255 (7.9%)
	Other	36 (1.1%)
	Mixed	35 (1.1%)
	Asian	31 (1.0%)
	Black	31 (1.0%)
	Latino	20 (0.6%)
	Indian	19 (0.6%)
	Irish Traveler	6 (0.2%)
	Arab	5 (0.2%)
**Program**	Space from Anxiety	1524 (45.7%)
	Space from Depression & Anxiety	987 (29.6%)
	Space from Depression	624 (18.7%)
	Space from Generalised Anxiety Disorder	76 (2.3%)
	To be confirmed**	121 (3.8%)
Treatment Expectations***	Extremely likely	90 (2.8%)
	Very likely	787 (24.5%)
	Somewhat likely	2021 (62.8%)
	Not very likely	199 (6.2%)
	Not at all likely	23 (0.7%)
Psychological Treatment****	Yes	440 (13.7%)
	No	2465 (82.5%)

* The breakdown of percentages does not sum to 100% because data was unavailable for some users

**This subset of users has been provided with the option of selecting their own program by their referring clinicians but have yet to confirm their choice

*** During sign-up clients were asked “How likely do you think SilverCloud will work for you?”

**** During sign-up clients were asked “Are you currently accessing any psychological treatment?”

Distributions of the scores on the primary outcome measures at baseline are provided in Fig. 3. 69% (69%) of users were at ‘caseness’ for depression (> 9 PHQ-9), and 79% for anxiety (> 7 GAD-7). Overall, 62% of users were at caseness for both, depression and anxiety at baseline. The majority of users (80.5%) exhibited moderate-to-severe general functional impairment as measured by the WSAS (see Table S1).

### Discharged users

Of the 3236 service users who activated an account between April 20th, 2021, and April 19th, 2022, 2255 were marked as ‘Discharged,’ and 714 remained in treatment. The statuses of the other accounts are detailed in Table S2. For the remaining sections of the results, we focus on the subset of users marked as ‘Discharged’ (n = 2255).

### Program usage

Analysis of all discharged users on an intention-to-treat basis revealed that the median number of logins was 8 (IQR, 3–18), the median number of minutes spent on the platform was 77.1 (IQR, 23–206), and the average number of supporter reviews received was 6.2.

When we focused on the subset of discharged users with at least two assessments (n = 1116), the median number of logins was substantially higher, at 17 (IQR, 10–28). The median number of minutes spent on the platform was 175.3 min, and the average number of supporter reviews received was 7.1. More details on these metrics can be found in Table S3 and Table S4.

### Clinical outcomes

**Fig. 4:**
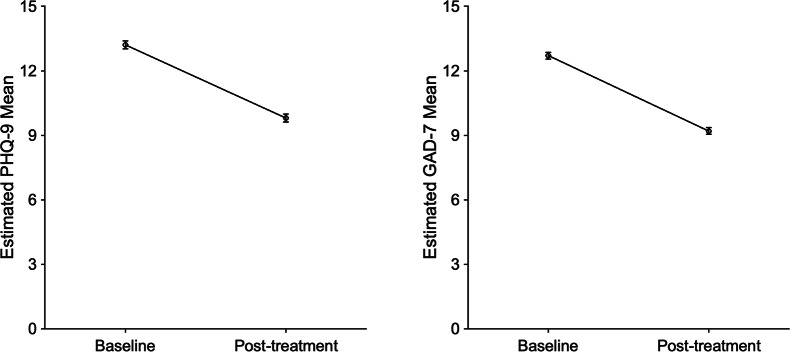
Estimated means at baseline and post-intervention for the PHQ-9 (A) and GAD-7 (B). Digital CBT was associated with significant reductions in scores on the PHQ-9 (*β* = 3.34, SE = 0.16, 95% CI [3.03, 3.65], *p* < 0.001, Cohen’s *d* = 0.85) and GAD-7 (*β* = 3.64, SE = 0.15, 95% CI [3.36, 3.93], *p* < 0.001, Cohen’s *d* = 0.99)

Linear mixed effects analyses on pre-to-post clinical scores for all discharged users (n = 2255) revealed that the digital CBT was associated with significant reductions in symptoms from baseline to post-intervention on both primary outcome measures of anxiety (*p* < 0.001) and depression (*p* < 0.001), with large effect sizes (see Fig. 4).

We additionally found that ‘time spent on the platform’ was an independent predictor of reduction in both depression (*p* < 0.001) and anxiety (*p* < 0.001) scores (adjusted for baseline scores), and the inclusion of this variable in the LMM significantly improved the goodness of fit of these models (PHQ-9 (*χ2* = 28.21, *p* < 0.001; GAD-7 (χ2 = 21.58, *p* < 0.001)), which is consistent with the well-established association between program usage and clinical outcomes [25].

Digital CBT was also associated with improvements in general functioning (*p* < 0.001), but the observed effect size was smaller (Cohen’s *d* = 0.33). ‘Time spent on the platform’ did not emerge as a significant predictor in this model (*p* = 0.061).

The total proportion of users that achieved recovery for either depression or anxiety was 49.7%, and the total proportion of users that achieved reliable improvement in either depression or anxiety was 57.5%. Of note, rates of reliable improvement increase as a function of baseline severity, with the highest rates of reliable improvement being observed in users with severe levels of depression and anxiety at baseline. In contrast, rates of recovery decrease as baseline severity increase, as users need a higher magnitude of change to meet the criteria for recovery (see Table 3).

Rates of reliable deterioration on these primary outcome measures were extracted for all users who completed at least two assessments (n = 1116). For the PHQ-9, the overall rate of reliable deterioration was 2.7%, and for the GAD-7 it was 4.3%.

**Table 3:** Rates of recovery, reliable improvement, no reliable change and reliable deterioration for each baseline severity level

	Baseline Severity	n	Recovery (n (%))	Reliable Improvement (n (%))	No Reliable Change (n (%))	Reliable Deterioration (n (%))
**PHQ-9**	Moderate	297	176 (59%)	89 (30%)	192 (65%)	16 (5%)
	Moderately Severe	254	98 (39%)	129 (51%)	118 (46%)	7 (3%)
	Severe	210	44 (21%)	117 (56%)	93 (44%)	0 (0%)
**GAD-7**	Moderate	296	176 (60%)	173 (59%)	107 (36%)	16 (5%)
	Severe	469	172 (38%)	308 (66%)	313 (33%)	5 (1%)

*Note: For the PHQ-9, Moderate corresponds to a score of 10–14, Moderately severe is 15–19, and Severe is 20–27; for the GAD-7, Moderate corresponds to a score of 10–14, and Severe is 15–21*

### User satisfaction

There were a total of 21,356 module ratings across all module evaluation questionnaires. The overall user satisfaction rating was 94.1% (percentage of all the ratings that were marked as “agree” or “strongly agree”), where 94.7% agreed the modules were interesting, 95.2% agreed the modules were relevant, 93% agreed the modules were helpful, and 93.3% agreed the program helps in making progress. A further breakdown of the ratings of these statements is provided in Table 4.

**Table 4:** Satisfaction ratings for each statement across all modules

	Strongly Agree	Agree	Disagree	Strongly Disagree
Interesting	1454 (27.2%)	3604 (67.6%)	223 (4.2%)	58 (1.1%)
Relevant	1909 (35.8%)	3176 (59.5%)	202 (3.8%)	52 (1.0%)
Helpful	1331 (24.9%)	3632 (68.1%)	309 (5.8%)	67 (1.3%)
Helping me make progress	1274 (23.9%)	3711 (69.5%)	293 (5.5%)	61 (1.1%)

## Discussion

This paper aimed to provide a one-year milestone evaluation of the digital CBT service provided through the national health service in Ireland. Specifically, it was of interest to gauge the demand and reach of the service through service-level data such as referrals and account activations and user-level data on demographics, baseline symptomatology, clinical outcomes, program usage and user satisfaction. Collectively, the evaluated service level and user level metrics corroborate the efficiency and effectiveness of the digital CBT service for providing evidence-based care to large numbers of people throughout Ireland. These findings further substantiate the burgeoning evidence supporting the real-world value of integrating digital CBT services into contemporary healthcare systems [22, 23, 26].

The sheer demand for the digital CBT service was apparent, with the number of referrals increasing monthly, culminating in 5,298 referrals within the year. The demand for the service was further corroborated by the volume of account activations, whereby the initial 12-month target of 1000 activated accounts was reached almost six months ahead of schedule, and a total of 3,227 accounts were ultimately activated within the year. General Practitioners (GPs) were the source of the majority of referrals. This is consistent with GPs constituting the largest group of healthcare professionals in primary care in Ireland and being the first point of contact for most individuals seeking professional help for a mental health problem [8, 44]. Notably, 993 (40%) out of an approximate total of 2,500 registered GPs[45] referred patients to the service, denoting a trend toward extensive reach of the service within the first year of it being available.

Most users had positive expectations about their potential to benefit from the digital CBT programs, with over 90% indicating that they believed the digital CBT was at least somewhat likely to work for them. This is a particularly important observation given the well-established relationship between expectations and treatment-related outcomes throughout medicine and psychotherapy (for a review, see [46]).

The data on program usage suggests a high level of engagement with the service, with the number of logins and the number of reviews received being higher than those reported in the IAPT services in the UK [20] and analogous to those reported in Mindspot a national digital CBT service implemented in Australia [47, 48]. User satisfaction ratings were also very high, exceeding 94% for overall satisfaction, consistent with those reported for Mindspot and another well-established national digital CBT service in Canada [32, 48, 49]. The program usage and user satisfaction data are therefore comparable to similar national initiatives that have been recognized as successful in other countries.

Overall, the digital CBT programs were associated with significant improvements in the primary outcome measures of depression and anxiety with large effect sizes (Cohen’s *d* > 0.8). The observation of a positive association between these clinical outcomes and the quantity of time spent on the platform is also promising as it provides support for the inference that the changes in clinical scores were related to participating in the programs as opposed to being explained by other epiphenomena. It is additionally encouraging to note that the overall rates of recovery observed in this evaluation (49.7%) are highly consistent with the rates of recovery reported in the most recent IAPT annual report, where it is reported that 50.2% of referrals that completed a course of treatment moved to recovery [50].

The digital CBT programs were also associated with significant improvements in general functioning, albeit the effect size was comparatively smaller. Given that it is conceivable that it could take some time for the CBT-related reductions in depression and anxiety to translate to improved general functioning, it will be of interest for future research to examine the durability of the improvements in depression and anxiety, as well as the longer-term changes in general functioning.

In terms of the demographics of the sample, while the distributions of reported education levels and the ethnicity of the users were highly consistent with the general population of Ireland [51], there was a notable gender imbalance in the sample. There were also some patterns in the distribution of the age groups that were not consistent with the demographics of the Irish population. Firstly, in terms of gender, almost three-quarters (72.9%) of users were female. This is highly discrepant with the most recent estimate of the male-to-female ratio of 9.8 to 10 in Ireland [52]. Such marked gender discrepancies have been reported consistently in similar naturalistic analyses of nationwide digital CBT initiatives. It has been suggested that they may be partially explained by a higher prevalence of depression and anxiety disorders in females and their higher treatment-seeking tendencies [53, 54]. Furthermore, Ireland has been identified as one of the countries with the most pronounced difference in the extent to which females versus males avail of mental healthcare in Europe [55]. This discrepancy warrants consideration of whether the mental healthcare needs of the male population in Ireland could be better supported through higher rates of use of this service by males and whether this could be facilitated by more targeted awareness campaigns for this cohort.

Regarding the reported age range of users, it is particularly noteworthy that only 2.3% of the sample were over the age of 65 years, despite this age cohort constituting 14.8% of the population of Ireland [56]. While lower uptake of digital health interventions amongst older adults has been reported elsewhere [57], it will be necessary for future work to disentangle the factors underlying this trend, including the extent to which it reflects age-related biases on the part of referring clinicians versus variables such as the internet or computer accessibility or capabilities on the part of older adults themselves.

One of the key forecasted benefits of technology-enabled interventions is that they should facilitate access to care for rural populations and those that frequently struggle with transportation issues [58]. The observation that there was a representation of service users from every county of Ireland provides a reasonable basis for assuming that the service has reached people residing in rural areas. Indeed, 18 accounts were activated from the most rural county in Ireland, Leitrim, wherein nine out of ten people live in rural areas [59]. Efforts to gain more precise insight into the rural versus urban representation, and the proportions of users who have mobility or travel limitations, should nonetheless be prioritized going forward such that a more informed characterization of the populations the service is catering for can be determined.

A noteworthy limitation of the present service evaluation is that the data to date do not allow for an accurate estimation or prediction of the actual demand for this service. This is primarily due to restricted resources for the first year of the partnership between the HSE and SilverCloud, which constrained the availability of account licenses and campaigns to promote awareness about the service. It is likely that future initiatives aimed at improving awareness of and promoting the service will reveal that the demand, uptake, and impact of the service will be manifold greater than the present data can reveal.

Furthermore, a feature of this service with notable room for improvement is the poor interoperability between the data systems that support referrals to the digital CBT platform. For instance, referring clinicians have been required to manually input patients’ email addresses and other personal details into a referral template when they make the referral. This places a non-trivial administrative burden on the clinicians. Further, since typographical errors are common with manual data entry [60], a proportion of patients that clinicians refer to the service never receive the intended email invite. Both the administrative burden and the scope for human errors to impact on the provision of care for patients could be mitigated in the future by integrating electronic health records (EHR) with the digital CBT platform in a manner that would facilitate the automated transmission of patient data directly from EHR when clinicians wish to refer a patient.

Finally, the observational nature of the present data does not allow for firm conclusions about the direct association between the intervention and the observed changes in clinical outcome measures. Accordingly, it should be acknowledged that changes in these measures could be at least partially attributable to extraneous variables, such as the known cyclical nature of mental health problems. That said, the *Space from Anxiety* and *Space from Depression* programs have previously been validated through a randomized control trial that included a wait-list control group [20], and positive associations between program usage and reductions in anxiety and depression symptoms were additionally observed in the present data. Taken together, these observations substantiate the clinical impact of the digital CBT programs.

Research examining the impact of concurrent pharmaceutical or psychological treatment, as well as the potential mediating and moderating impact of several other baseline characteristics, should be a priority for future work in this area. Developments from this line of inquiry will be particularly important for predicting treatment response and guiding more precise and personalised treatment allocation.

In summary, the findings from this one-year milestone evaluation demonstrate how evidence-based digital CBT can be provided at scale and lead to symptom reductions with large effect sizes for patients seeking help for depression and anxiety. The nationwide reach of the service also demonstrates the viability of the digital medium to extend care to distal geographic regions and reduce the mental healthcare burden of under-resourced health services. These findings provide clear justification for the continued support of this service in primary care in Ireland and the more widespread implementation of similar services in other international public healthcare settings.

### Electronic supplementary material

Below is the link to the electronic supplementary material.

Supplementary Material 1

Supplementary Material 2

## Data Availability

The dataset, along with the data analysis scripts, for the current study are available on reasonable request.

## Abbreviations

HSE: Health Service Executive
SCH: SilverCloud Health
IAPT: Improving Access to Psychological Therapies
GP: General Practitioner
CBT: Cognitive Behavioral Therapy
PHQ-9: Patient Health Questionnaire-9
GAD-7: Generalised Anxiety Disorder-7
WSAS: Work and Social Adjustment Scale
ITT: Intention-to-Treat
LMM: Linear Mixed-Effects Model
RCI: Reliable Change Index
