# Supplementary material for: Implementing digital mental health interventions at scale: one-year evaluation of a national digital CBT service in Ireland
Source: Int J Ment Health Syst. 2023 Oct 10;17:29. doi: 10.1186/s13033-023-00592-9 (PMC10563351; doi:10.1186/s13033-023-00592-9)
Supplement: Supplementary file 2 — Supplementary Material 2 [file 13033_2023_592_MOESM2_ESM.pdf]

Implementing digital mental health interventions at scale: One-year evaluation of a national digital CBT service in Ireland

Supplementary Materials

Risk Management Protocol

Risk is continuously monitored via the suicidality question of the PHQ-9 (i.e. item 9). If a user reports a score of 2 or more on item 9 of the PHQ-9, the P4 screener (Dube et al., 2010) is automatically triggered. The P4 screener asks qualifying questions to obtain a more informed understanding of the level of risk and suitability for the program. If the users' answers to any of the P4 screener questions qualify as high risk, the service user will be automatically excluded and informed that they will receive a phone call from the clinical supervisor within 48 hours. They are also directed to the SCH crisis support centre. Supporters are also trained to monitor and report potential concerning content shared by users. If supporters identify risk they must complete a risk report and risk log, and they must additionally inform the clinical supervisor. The clinical supervisor must then review the risk report within 48 hours, and decide if the service user requires a phone call assessment. If, during the phone call, the service user is deemed unsuitable to continue with the program the clinical supervisor will update the risk report and risk log. SCH will also inform the referrer of the risk and ask for an additional assessment.

Table S1. Baseline levels of depression, anxiety and general impairment as measured by the PHQ-9, GAD-7, and WSAS respectively.

	Baseline Severity Category	n (%)	Caseness n (%)	Overall Mean (SD)
**PHQ-9**	Minimal (0-4)	232 (7.2%)	2122 (68.5%)	13.3 (6.2)
	Mild (5-9)	745 (23.0%)		
	Moderate (10-14)	796 (24.6%)		
	Moderately Severe (15-19)	745 (23.0%)		
	Severe (20-27)	581 (18.0%)		
	Unavailable	137 (4.2%)		
**GAD-7**	Minimal (0-4)	182 (5.6%)	2428 (78.6%)	12.8 (5.3)
	Mild (5-9)	771 (23.8%)		
	Moderate (10-14)	839 (25.9%)		
	Severe (15-21)	1296 (40.0%)		
	Unavailable	148 (4.6%)		
**WSAS**	Low Impairment (0-9)	455 (14.1%)	2605 (85.1%)	18.0 (8.2)
	Moderate Impairment (10-21)	1316 (40.7%)		
	Severe Impairment (20-40)	1289 (39.8%)		
	Unavailable	176 (5.4%)		

Status of Accounts

Of the 3227 service users who activated an account between April 20th, 2021 and April 19th, 2022, 2255 have now been 'Discharged,' and 714 are currently in treatment. A further 194 accounts are 'Pending' (i.e. the service user has not yet completed the sign-up process) and six are categorised as 'New Referral' (i.e. the service user has completed the sign-up process and is waiting to be assigned a supporter). The remaining accounts have either been 'Excluded' (i.e. the service user has been excluded due to various reasons, including a small number cases where suicide risk was flagged), 'Deleted' (i.e. the service user has requested for their information to be deleted), or 'Paused' (i.e. the service user has requested to temporarily

suspend their treatment). For a more detailed breakdown of the number of accounts under each of these statuses please see Table S2.

Table S2. Status of accounts

Discharged	Deleted	Excluded	Current	Pending	Paused	New Referral
2255 (69.6%)	8 (0.3%)	57 (1.8%)	714 (22.0%)	194 (6.0%)	2 (0.1%)	6 (0.2%)

Table S3. Program usage metrics for all discharged users (n=2255)

	Mean (SD)	Min - max	Median
**Number of logins**	14.6 (20.1)	1 – 251	8.0 (3-18)
**Number of reviews**	6.2 (1.5)	1 – 10	6.0 (5-8)
**Length of use (minutes)**	171.2 (267.6)	2.7 – 2972.6	77.1 (23-206)
**Time per session (minutes)**	12.3 (11.0)	0.8 – 131.4	9.3 (5.6-15.3)

Table S4. Program usage metrics for discharged users with at least 2 assessments (n=1116)

	Mean (SD)	Min - max	**Median** (IQR)
**Number of logins**	23.4 (22.9)	2 – 245	17.0 (10-28)
**Number of reviews**	7.1 (1.1)	2 – 10	7.0 (6-8)
**Length of use (minutes)**	279.4 (329.4)	4.6 – 2972.6	175.3 (85.4-355)
**Time per session (minutes)**	12.4 (8.9)	1.5 – 91.7	10.2 (6.4-15.7)
